# Comparison of NIRS exercise intensity thresholds with maximal lactate steady state, critical power and rowing performance

**DOI:** 10.5114/biolsport.2024.129486

**Published:** 2023-11-20

**Authors:** Leonardo Trevisol Possamai, Fernando Klitzke Borszcz, Rafael Alves de Aguiar, Ricardo Dantas de Lucas, Tiago Turnes

**Affiliations:** 1 Physical Effort Laboratory, Sports Center, Federal University of Santa Catarina, Florianópolis, Brazil; 2 Human Performance Research Group, Center for Health and Sport Science, Santa Catarina State University, Florianópolis, Brazil

**Keywords:** Near-infrared spectroscopy, Physiological markers, Exercise intensity domains, Muscle oxygenation, Sports performance

## Abstract

This study aimed to compare the intensity of deoxygenated haemoglobin concentration ([HHb]) and tissue saturation index (TSI) breakpoints ([HHb]-BP and TSI-BP) with maximal lactate steady state (MLSS) and critical power (CP), and to describe their association with 2000-m rowing ergometer performance. Fourteen male rowers performed on a rowing ergometer: I) a discontinuous incremental test with 3-min stages (INC_3_); II) a continuous incremental test with 1-min stages (INC_1_); III) constant workload tests to determine MLSS; and IV) performance tests of 500 m, 1000 m, 2000 m and 6000 m to determine CP.CP (257 ± 39 W; 3.79 ± 4.1 L · min^−1^) was higher than [HHb]-BP_3_ (205 ± 26 W; 3.48 ± 2.9 L · min^−1^), [HHb]-BP_1_ (207 ± 27 W; 3.27 ± 3.2 L · min^−1^), and TSI-BP_3_ (218 ± 31 W; 3.51 ± 3.0 L · min^−1^), but not higher than TSI-BP_1_ (222 ± 34 W; 3.43 ± 3.2 L · min^−1^). MLSS (187 ± 26 W; 3.33 ± 3.2 L · min^−1^) was lower than TSI-BP_3_ and TSI-BP_1_ for power output, but not different in any comparison for ⩒O_2_. The limits of agreement for power output and ⩒O_2_ suggest poor agreement among these thresholds. The low level of agreement compromises the use of [HHb]-BP and TSI-BP for estimating MLSS and CP; therefore, these thresholds should not be considered interchangeable.

## INTRODUCTION

Maximal lactate steady state (MLSS) and critical power (CP) are the most utilized exercise intensity thresholds for heavy-severe domain boundary demarcation [1]. However, their determination is laborious, making their incorporation in the routine assessment of athletes difficult. As an alternative to time-consuming protocols, single-day exercise intensity thresholds have important applications to estimate MLSS and CP [2, 3]. For this purpose, there are several techniques based on blood lactate concentration (BLC), heart rate, and ventilatory responses determined in incremental exercise testing [2, 3]. However, these thresholds are protocol-dependent, present large intra-individual variability, and in most cases overestimate MLSS, especially during rowing exercise [3, 4].

In addition to these techniques, near-infrared spectroscopy (NIRS) has been proposed as a novel and non-invasive tool to estimate exercise thresholds in different exercise modes [5, 6, 7]. This technique determines exercise intensity thresholds through the changes in deoxygenated haemoglobin (HHb) and myoglobin concentrations [7] or the tissue saturation index (TSI) [5]. Changes in [HHb] and TSI provide a reliable estimate of the dynamic balance between O_2_ supply and O_2_ consumption, although the latter may be more influenced by cutaneous blood flow [8, 9]. In contrast, TSI is determined using a spatially resolved method (called multi-distance spectroscopy) and, therefore, reflects a greater volume of the muscle of interest. During incremental exercise tests, [HHb] and TSI signals exhibit a breakpoint (i.e., [HHb]-BP and TSI-BP, respectively) which represents an attenuation in the response and is reported as a possible exercise threshold [5, 7]. However, while the protocol dependence of exercise thresholds (e.g., based on BLC) is well-documented, the influence of the incremental test in [HHb]-BP and TSI-BP during rowing exercise is still uncertain.

Due to its relationship with the whole-body physiological responses, [HHb]-BP and TSI-BP have been compared to other physiological-derived exercise intensity thresholds, especially in cycling [6, 10–13] and running exercises [5]. For cycling, there were no mean differences for the ⩒O_2_ at [HHb]-BP and MLSS [1, 10, 11] or [HHb]-BP and CP [1, 11]. On the other hand, power output was higher at [HHb]-BP when compared to MLSS and CP [1, 11]. During running exercise, MLSS and TSI-BP presented similar average speeds [5]. For rowing exercise, however, less evidence is available. Turnes et al. [7] reported that the power output associated with [HHb]-BP was not significantly different from the fixed BLC of 3.5 mmol · l^−1^ in trained rowers, which in turn overestimated MLSS in rowing [3]. Accordingly, comparisons of NIRS-derived thresholds with single-day thresholds derived from BLC, HR, or ventilatory responses may not be informative because they overestimated MLSS [3, 4, 14] and underestimated CP [3] in rowing. Thus, a direct comparison of [HHb]-BP and TSI-BP with MLSS and CP remains to be performed in rowers.

Therefore, the aim of this study was twofold: 1) to determine the relationship and the agreement of [HHb]-BP and TSI-BP derived from two incremental tests with MLSS and CP, and 2) to verify the relationship between NIRS-derived thresholds with 2000-m rowing ergometer performance. Based on the outstanding difference between MLSS and CP during rowing exercise, it was hypothesized that NIRS-derived thresholds would overestimate and underestimate MLSS and CP, respectively.

## MATERIALS AND METHODS

### Participants

Fourteen regional- and national-level male rowers (mean ± standard deviation [SD]; age: 26 ± 13 years; height: 1.82 ± 0.05 m; body mass: 81.0±7.6 kg; training experience: 4.3±3.0 years) volunteered and provided written informed consent (or it was provided by a relevant parent, as one participant was under the age of 18) to participate in this study. The participants were scull (four) and sweep (five) rowers, five lightweight and four heavyweight. This study was approved by the Institutional Ethics Committee for Research on Human Subjects (number 3.191.968) and was performed according to the Declaration of Helsinki. Rowers trained 6 days a week, including 525 ± 183 min per week of on-water and indoor rowing training as well as resistance workouts. The study was performed during the season, in a training period of preparation for the national evaluation system.

### Design

Each rower completed 8–10 testing sessions within 30 days. All tests were interspersed with ≥ 48 h of recovery. The rowers accomplished: I) a discontinuous step incremental test with 3-min exercise stages and 30-s recovery intervals (INC_3_) and a continuous incremental test with 60-s exercise stages (INC_1_); II) two to four visits to determine MLSS; and III) three to four maximal time-trial tests of 500 m, 1000 m, 2000 m, and 6000 m to determine CP and the finite work capacity above CP (*W*’). Each participant was always tested at the same time of day (± 2 h) in a temperature-controlled laboratory (22 ± 1 °C). Performance tests were conducted in the athletes’ clubs following standardized protocols. Athletes were instructed to abstain from vigorous physical activity for 24 h before each test and maintain their usual routine during the testing period.

### Materials

All tests were performed on an air-braked rowing ergometer (Concept 2E, Morrisville, VT, USA). Rowers individually and manually set the drag factor on the ergometer on their first visit (mean ± standard deviation: 125 ± 3 arbitrary units). Oxygen uptake (⩒O_2_) was monitored using an automated open-circuit breath-by-breath gas analyser (Quark CPET; Cosmed, Rome, Italy) calibrated with known concentration values of oxygen and carbon dioxide. Capillary blood samples (25 µL) were taken from the earlobe and analysed for BLC using enzyme electrode technology (YSI 2700, Yellow Springs, USA). The NIRS signals were obtained from the vastus lateralis muscle using portable multi-distance continuous wave spectroscopy (PortaMon; Artinis Medical Systems BV, Zetten, The Netherlands).

### Exercise Tests

#### Incremental tests

On two separate days, rowers performed a continuous and a discontinuous incremental test until voluntary exhaustion to determine maximal oxygen uptake (⩒O_2max_) and NIRS-derived thresholds. During the discontinuous incremental test (INC_3_), the initial workload was set at 130 W for 3 min and then increased by 30 W every 3 min [15]. At the end of each stage, a 30-s rest period was provided to collect blood samples. During the continuous incremental test (INC_1_), after a 2-min baseline period at 115 W, the initial PO was set at 130 W for 1 min and then increased by 15 W every 1 min [16]. The peak power output (Ppeak) in the INC_3_ and INC_1_ was defined as the workload attained at exhaustion, when the test was completed at the end of the stage. If the test was concluded before the final stage had been completed, the Ppeak was calculated as previously described [17]. The ⩒O_2max_ was taken as the highest 15-breath rolling average value reached by ⩒O_2_ [18]. To analyse ⩒O_2_ throughout the incremental tests, the average of the final 30 s of each stage was computed [7].

### Maximal lactate steady state determination

For the determination of MLSS, 2 to 4 constant work rate submaximal tests lasting 30 min were performed on different days. Before each test, a moderate-intensity warm-up of 5 min was executed followed by a 5-min passive recovery period. The workload for the first trial corresponded to 70% Ppeak from the INC_3_ and was then increased or reduced in 5% until MLSS determinarion [14]. Capillary blood samples were collected at the pre-test and the end of ten and thirty minutes, during a 30-s rest period. The MLSS was defined as the highest workload where BLC did not increase by more than 1 mmol·l^−1^ between the 10^th^ and 30^th^ minutes of the test [19]. The ⩒O_2_ value of MLSS was calculated by the average value of the final minute of exercise.

### Critical power determination

CP and *W*’ were estimated from three to four predictive trials, which were performed in random order [3]. Time-trial performance tests of 500 m, 1000 m, 2000 m, and 6000 m were carried out [3]. One rower was unable to finish the tests, and 9 and 4 rowers completed the 500-, 1000-, and 2000-m and 500-, 2000- and 6000-m tests, respectively, following the distances selected by the schedule in their clubs.

CP and *W*’ were calculated according to the linear work-time (*P* = [*W*’/time] + CP), linear inverse-of-time (*P* = *W*’ [1/time] + CP), and hyperbolic (time = *W*’/[*P* – CP]) models, then three values of CP and *W*’ estimates for each subject were provided. Data from the linear work-time model were used for subsequent analyses due to a better fit of linear data (R^2^ = 0.998 ± 0.002) and the lowest standard error for CP (4.2% ± 2.6%). The ⩒O_2_ value of the CP was estimated by linear regression analysis from the relationship between the power output and ⩒O_2_ from the INC_3_ (R^2^ = 0.945 ± 0.033).

### Data Analysis

#### NIRS procedures

The NIRS apparatus was used to detect relative changes in local muscle [HHb] and TSI. The raw data were recorded at 1 Hz. The NIRS probe was placed on the vastus lateralis belly approximately halfway between the trochanter and knee joints after the skin area had been shaved and wiped. Skinfold thickness was measured at the site of NIRS application (12.8 ± 5.4 mm) using a Lange Skinfold Caliper (Incorporated Cambridge, Maryland, USA), to ensure that the adipose thickness did not substantially contaminate muscle NIRS signals [9]. The device was covered with a black light-absorbing cloth to prevent contamination from ambient light.

### NIRS-derived thresholds

The average of the raw data from the final 10-s of each stage during the incremental tests was used to individually plot [HHb] and TSI as a function of the workload. The [HHb]-BP and TSI-BP were determined by fitting the following double-linear model to the data [7, 20]:


$$\begin{array}{c} f=if\left[x<BP,g(x),h(x)\right]\\ g(x)=i_{1}+(s_{1}x)\\ i_{2}=i_{1}+(s_{1}BP)\\ h(x)=i_{2}+\left[s_{2}(x-BP)\right]\\ fifty \end{array}$$

Where *f* is the double-linear function, × is the workload, and *y* is [HHb] or TSI, *BP* is the time coordinate corresponding to the interception of the two regression lines (i.e., [HHb]-BP and TSI-BP), *i*_1_ and *i*_2_ are the intercepts of the first and second linear function, respectively, and *s*_1_ and *s*_2_ are the slopes. After the fitting, the [HHb]-BP and TSI-BP were identified as the inflection point followed by a plateau, under visual inspection by two independent researchers. The fitting was employed to enable visual inspection and to improve objectivity in data analysis, as confirmed by no disagreement between investigators. The two thresholds were determined during the INC_3_ ([HHb]-BP_3_ and TSI-BP_3_) and INC_1_ ([HHb]-BP_1_ and TSI-BP_1_).

### Statistical analysis

Descriptive data are presented as mean ± SD. Statistical parameters are reported as mean point estimates with 95% confidence intervals (95% CI). Paired-sample Student *t*-tests were used to compare incremental test variables. The relationships between NIRS-derived thresholds with MLSS, CP, and 2000-m performance time were evaluated through a simple linear regression analysis, reporting the Pearson’s coefficient of correlation (r), and the typical error of the estimate (TEE) [21]. Agreements between NIRS-derived thresholds with MLSS and CP were analysed using Bland-Altman’s graphical analysis [22].

Repeated measures mixed effects ANOVA models were used for comparisons between NIRS-derived thresholds with MLSS and CP (power output and ⩒O_2_). Fixed effects were the exercise intensity thresholds and the random effect was the identity of each subject.

When appropriate, Dunnett’s post-hoc test was used to identify pairwise differences. Correlation coefficients were interpreted as follows: < 0.09 trivial, 0.10 to 0.29 small, 0.30 to 0.49 moderate, 0.50 to 0.69 large, 0.70 to 0.89 very large, 0.90 to 0.99 nearly perfect, and 1.0 perfect [21]. Statistical significance was set at p < 0.05.

## RESULTS

**FIG. 1:**
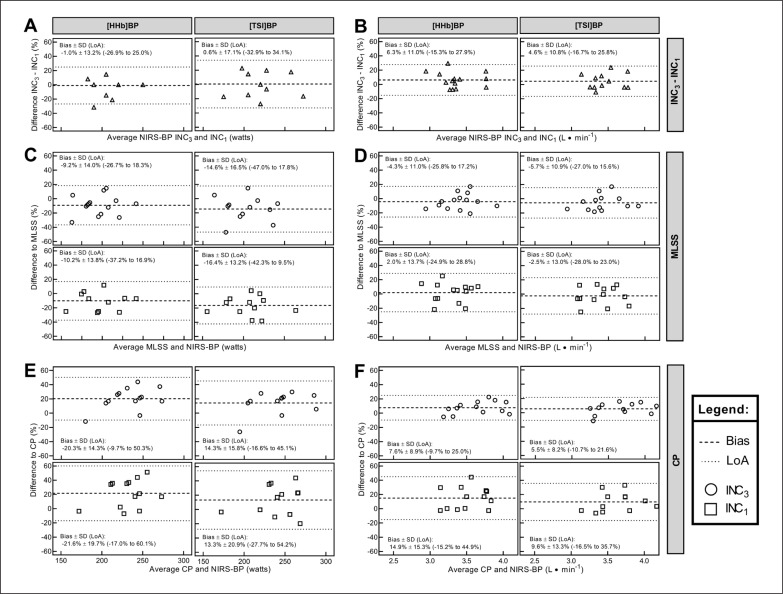
Bias and limits of agreement between NIRS-derived thresholds determined in different incremental tests (A-B), maximal lactate steady state (C-D), and critical power (E-F).

During the incremental tests, there were no differences in Ppeak (INC_3_: 308 ± 39; INC_1_: 311 ± 37 W; p = 0.369), ⩒O_2max_ (INC_3_: 4.19 ± 4.0; INC_1_: 4.09 ± 4.9 mL · min^−1^; p = 0.106), and the power output and ⩒O_2_ at [HHb]-BP (p = 0.765 and 0.054, respectively), and TSI-BP (p = 0.910 and 0.174, respectively) (Figure 1A-B). In two participants, the TSI-BP was not detectable, as their profiles did not display any clear breakpoint.

Comparisons between the different NIRS-derived thresholds from the same INC revealed that [HHb]-BP_3_ and TSI-BP_3_ were significantly different for power output (p = 0.018; bias ± LoA = 5.5% ± 14.1%) but not for ⩒O_2_ (p = 0.350; bias ± LoA = 1.2% ± 8.7%). There were no significant differences between [HHb]-BP_1_ and TSI-BP_1_ for power output (p = 0.182; bias ± LoA = 5.9% ± 29.5%) and ⩒O_2_ (p = 0.148; bias ± LoA = 3.6% ± 16.3%). The correlation for power output of [HHb]-BP_3_ with TSI-BP_3_ was very large (r = 0.87; 0.61 to 0.96) and that for [HHb]-BP_1_ with TSI-BP_1_ was moderate (r = 0.45; -0.13 to 0.80). Regarding ⩒O_2_, the correlation was very large (r = 0.87; 0.61 to 0.96) and large (r = 0.58; 0.04 to 0.86) between [HHb]-BP_3_ with TSI-BP_3_ and [HHb]-BP_1_ with TSI-BP_1_, respectively.

The means ± SD of MLSS, CP, and NIRS-derived thresholds for power output and ⩒O_2_ are shown in Table 1, and individual data are attached as supplementary material. There were significant differences between NIRS-derived thresholds and MLSS for power output (F_(2.7, 33.4)_ = 5.174; p = 0.006), but not for ⩒O_2_ (F_(2.7, 33.7)_ = 2.068; p = 0.130). The post hoc analysis revealed that power output at MLSS was significantly lower than power output at TSI-BP_3_ and TSI-BP_1_ (p = 0.018 and 0.003, respectively), but not at [HHb]-BP_3_ and [HHb]-BP_1_ (p = 0.090 and 0.053, respectively).

**TABLE. 1:** Descriptive values of exercise intensity thresholds for power output and oxygen uptake.

	[HHb]-BP_1_	[HHb]-BP_3_	TSI-BP_1_	TSI-BP_3_	MLSS	CP
Power output (W)	207 ± 27	205 ± 26	222 ± 34	218 ± 31	187 ± 26	257 ± 39
Power output (%Ppeak)	67.2 ± 8.8	67.0 ± 6.5	71.4 ± 9.7	70.6 ± 8.9	60.9 ± 4.1^*^	82.6 ± 9.0^*^
⩒O_2_ (L · min^−1^)	3.27 ± 3.2	3.48 ± 2.9	3.43 ± 3.2	3.51 ± 3.0	3.33 ± 3.2	3.79 ± 4.1
⩒O_2_ (%⩒O_2max_)	80.9 ± 11.3	83.3 ± 8.0	83.7 ± 9.9	84.5 ± 7.4	79.7 ± 6.3^*^	89.7 ± 8.7^*^

Data are shown as mean ± SD. CP: critical power; MLSS: maximal lactate steady state; [HHb]-BP_1_: deoxygenated haemoglobin breakpoint during incremental test with 1 min stages; [HHb]-BP_3_: deoxygenated haemoglobin breakpoint during incremental test with 3 min stages; TSI-BP_1_: tissue saturation index breakpoint during incremental test with 1 min stages; TSI-BP_3_: tissue saturation index breakpoint during incremental test with 3 min stages;

* : significant different from breakpoints determined during incremental test with 3 min stages (p < 0.05).

For CP, there were significant differences in power output (F_(2.6, 31.4)_ = 8.342; p = 0.001) and ⩒O_2_ (F_(2.2, 27.4)_ = 6.365; p = 0.004). Power output and ⩒O_2_ at CP were significantly higher than at [HHb]-BP_3_ (p = 0.001 and 0.017, respectively), [HHb]-BP_1_ (p = 0.007 and 0.014, respectively), and TSI-BP_3_ (p = 0.008 and 0.027, respectively), but not significantly higher than at TSI-BP_1_ (p = 0.101 and 0.069, respectively). The agreement analyses between MLSS/CP and NIRS-derived thresholds are presented in Figure 1C-F.

**FIG. 2:**
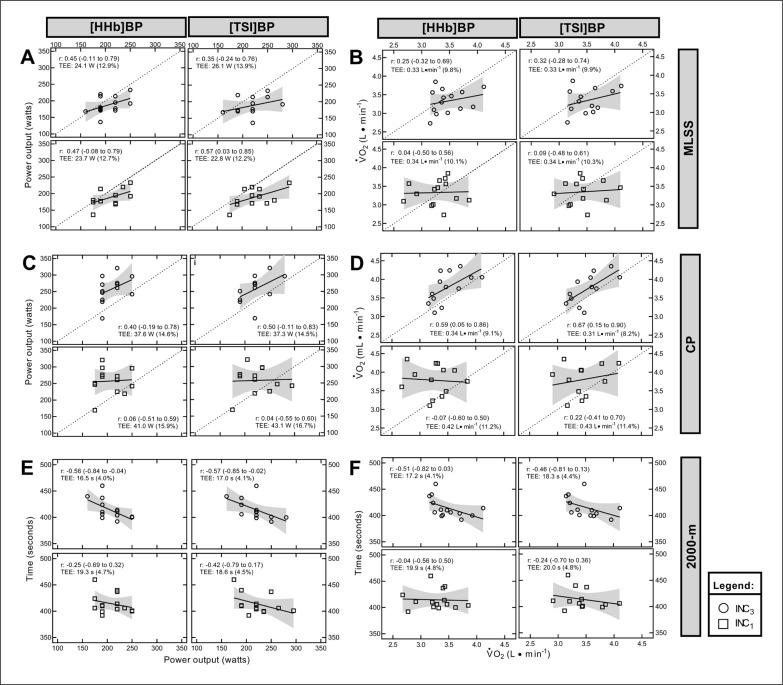
Relationship between maximal lactate steady state (A-B), critical power (C-D), and 2000-m performance (E-F) with NIRS-derived thresholds.

Figure 2 presents the linear regression between the power output and ⩒O_2_ at MLSS (Panels A and B), CP (Panels C and D), and 2000-m performance time (panels E and F) with the power output and ⩒O_2_ at NIRS-derived thresholds. Trivial to large correlations occurred among MLSS and CP with NIRS-derived thresholds. For 2000-m performance time, trivial to large correlations were observed.

## DISCUSSION

In accordance with the primary hypothesis, poor agreement and an association of MLSS/CP with NIRS-derived exercise intensity thresholds were observed. Although such threshold was previously compared with the anaerobic threshold during rowing exercise [7], the novel aspect observed herein was the direct comparison with MLSS and CP. These two markers are important metabolic thresholds, with MLSS occurring at a lower intensity than CP, mainly in rowing exercise [3]. Despite this, the utilization of this novel approach for estimating MLSS and CP during rowing ergometer exercise is questionable because of the clear disagreement observed.

Regarding MLSS, no statistically significant differences in any NIRS-derived thresholds for ⩒O_2_ and or both [HHb]-BPs for power output were found. Nevertheless, random errors expressed as LoA values were quite high and the association was low, which prevents us inferring that NIRS-derived thresholds are interchangeable with MLSS. Previous investigations compared NIRS-derived thresholds and MLSS in running [5] and cycling [1, 10], the former using TSI-BP in the gastrocnemius [5] and the latter [HHb]-BP in the vastus lateralis [1, 10]. Likewise, the studies showed significant differences in the means and considerable errors of agreement between the running speed at MLSS and TSI-BP (bias ± LoA = ~3.5% ± 6.0%) [5] and the ⩒O_2_ at MLSS and [HHb]-BP (bias ± LoA =~-0.7% ± 22.6%) [10]. Although direct comparisons among studies should be carefully analysed because of differences in exercise modes, muscles involved, or methodological approaches, these findings demonstrate that TSI-BP and [HHb]-BP present high variability and should not be considered equivalent to MLSS.

With regard to CP, the present study verified that all NIRS-derived thresholds were lower than CP intensity, except for TSI-BP_1_. In addition to the evident mean differences for power output and ⩒O_2_, the LoA values suggest poor agreement between these thresholds and CP. As observed for MLSS, the comparison of NIRS-derived thresholds with CP is limited for cycling exercise and [HHb]-BP [1, 11, 23]. Comparisons with TSI-BP are still incipient. In healthy individuals, [HHb]-BP workload was higher than CP in two different studies [11, 23]. Of note, these studies estimated CP from the 3-parameter hyperbolic model [11] or 2-parameter best individual fit model before and after six weeks of training intervention [23]. In contrast, the present result indicated that [HHb]-BP_3_ and [HHb]-BP_1_ workloads were lower than CP for rowing. Despite the well-known protocol dependencies that influence the CP workload, it was previously observed that CP was ~30% higher than MLSS in rowing [3], a much higher difference than observed in cycling (~10%) [24], which in part may explain these contrasting findings. Finally, it is important to note that the predicted ⩒O_2_ at CP from the INC_3_ may still underestimate the real ⩒O_2_ at CP due to the inherent ⩒O_2_ slow component at this intensity. This would result in an even greater ⩒O_2_ mean difference between NIRS-derived thresholds and CP than that reported in this study.

The rowing motor pattern is a complex task, which may make it difficult to determine the association between systemic and local responses due to the heterogeneity in the blood flow and O_2_ extraction responses among muscles [25]. Therefore, as NIRS devices provide a signal of muscle oxygenation only at the level of a probe, and rowing exercise recruits several muscles associated with coordination of upper and lower limbs, caution is necessary when adopting [HHb]-BP or TSI-BP as an index presenting correspondence with different markers of systemic responses.

Some previous investigations verified the protocol dependence for workloads at thresholds derived from different incremental tests [2, 3, 4]. Conversely, the present study verified no differences, but high random error between NIRS-derived thresholds from the INC_3_ and INC_1_, suggesting that NIRS-derived thresholds cannot be used interchangeably. Interestingly, the determination of NIRS-derived thresholds has been assessed mainly in ramp incremental tests [1, 11, 23], which is not feasible to perform on a rowing ergometer. In an attempt to get closer to a ramp incremental test, the present study used a short step test (i.e., INC_1_). However, the 30 seconds rest in INC_3_ allows a partial recovery, which probably could influence the physiological pattern of NIRS-variables compared to INC_1_. Furthermore, the associations between [HHb]-BP and TSI-BP and between NIRS-derived thresholds and MLSS and 2000-m performance were much lower when using the NIRS-derived thresholds from the INC_1_. Thus, the use of the INC_3_ is suggested in future studies in order to analyse the physiological relevance of NIRS-derived thresholds in rowing.

For cycling, running and swimming the mean differences between MLSS and CP (or critical velocity) ranged from 4% to 16%, and recently Galán-Rioja et al. [24] reported a mean difference of ~11% in cycling exercise, from a meta-analysis. Thus, it is reasonable to assume that CP occurs at greater intensities than MLSS at least for trained individuals. Furthermore, the substantial mean difference of approximately 30% between MLSS and CP reported before [3] is probably the largest difference found in the literature. The possible reason is the lower relative values of the MLSS in rowers [4, 14, 26] due to a different pattern of blood lactate dynamics. Indeed, Beneke et al. [27] verified that MLSS seems to decrease with the increase of primarily engaged muscle mass, which may influence the results considering the motor pattern of rowing and the muscle mass engaged. Therefore, the previous findings highlighted that CP and MLSS occur at different physiological intensities and cannot be considered analogous. Finally, the novelty of the present study was the comparison between NIRS-derived thresholds and both MLSS and CP in trained rowers.

The NIRS device can deliver real-time feedback to athletes and monitoring the [HHb] and TSI may be interesting during a training session in order to adjust the intensity, quantify the workload, and provide specific information about the working muscles [5, 28, 29, 30]. This could be even more relevant for on-water rowing, in which monitoring of training is more complex than indoor rowing. The small difference of [HHb]-BP with the fixed BLC of 3.5 mmol·l^−1^ and the good association with 2000-m rowing ergometer performance initially suggested that the local index may distinguish rower performance [7]. In the present study, [HHb]-BP and TSI-BP had a large and very large correlation with 2000-m rowing ergometer performance, respectively. The difference between the magnitude of correlation may be related to the increased tissue volume under consideration in the TSI compared to [HHb], considering that the vastus lateralis volume largely explained the variance in rowing ergometer performance [31]. In contrast, the TSI-BP was not detectable in all athletes and only captures a small portion of the selected muscle. Therefore, caution is required when establishing a link between NIRS-exercise thresholds and systemic responses. To date, regarding the performance prediction, the incremental test peak power output remains the most important predictor of 2000-m rowing performance at different training levels [3, 15, 32, 33], which may represent a more practical and easier marker than the NIRS-derived thresholds.

## CONCLUSIONS

The NIRS-derived thresholds presented poor agreement with MLSS and CP, compromising their interchangeable use for estimating the markers of exercise intensity domains. Thus, the NIRS-derived thresholds are not useful indices for rowers.
